# An Atypical Presentation of Vascular Injury in Extremity Trauma Beyond Current Guidelines

**DOI:** 10.7759/cureus.90413

**Published:** 2025-08-18

**Authors:** Taylor Broudy, Logan Gonsalves, Jonathan King, Kakra Hughes, Alexander Evans

**Affiliations:** 1 Department of Surgery, Howard University College of Medicine, Washington, D.C., USA; 2 Department of Surgery, Howard University Hospital, Washington, D.C., USA; 3 Department of Surgery, Division of Vascular Surgery, Howard University Hospital, Washington, D.C., USA; 4 Department of Surgery, Division of Trauma and Acute Care Surgery, Howard University Hospital, Washington, D.C., USA

**Keywords:** ankle-brachial index, arteriovenous fistula, computed tomography angiography, extremity trauma, vascular injury

## Abstract

CT angiography (CTA) is a key diagnostic tool for evaluating vascular injuries in trauma patients. Current guidelines from the Eastern Association for the Surgery of Trauma (EAST) recommend CTA when there are abnormal physical findings or an ankle-brachial index (ABI) ≤ 0.9. We present the case of a 32-year-old male with a gunshot wound to the thigh who had a normal ABI and did not meet the established criteria for imaging. However, CTA was performed due to subtle signs, including distal limb swelling, and revealed a traumatic femoral arteriovenous fistula. The patient subsequently underwent successful endovascular repair. This case highlights the limitations of existing guidelines and underscores the continued importance of a thorough physical examination, a high index of suspicion, and sound clinical judgment.

## Introduction

Guidelines published by the Eastern Association for the Surgery of Trauma (EAST) recommend obtaining CT angiography (CTA) for hemodynamically stable patients with extremity trauma who demonstrate soft signs of vascular injury and/or an abnormal ankle-brachial index (ABI) [1]. When hard signs of vascular compromise are present, the guidelines recommend proceeding directly to the operating room or interventional suite without imaging [1,2]. Guidelines from the American Association for the Surgery of Trauma and the World Society of Emergency Surgery concur with EAST, emphasizing that the decision to pursue further imaging should be based on physical examination findings and recommending against CTA when the ABI is greater than 0.9 [2].

Research has also suggested that CTA may be overutilized in the evaluation of extremity trauma [3,4]. However, some diagnoses, such as traumatic arteriovenous fistula (AVF), defined as an abnormal connection between an artery and an adjacent vein when both vessels are simultaneously injured in penetrating trauma, may be missed due to atypical presentations [5,6]. It is estimated that diagnosis is delayed in approximately 70% of patients with AVF because of misleading physical examination findings, with delays extending over a decade in some cases [6-8].

In the absence of vascular injury indicators on physical examination, an abnormal ABI (≤0.9) does warrant further evaluation with CTA. However, an abnormal ABI is not always present in patients with vascular injury. For this reason, subtle findings, such as distal swelling, should not be overlooked, as they may indicate the need for CTA when classical signs of arterial injury are absent and the ABI is normal. We report a case of a traumatic femoral AVF secondary to a gunshot wound across the distal thigh in a patient with normal peripheral pulses and an ABI >0.90 on the affected side, and we discuss the clinical indication that prompted CTA.

## Case presentation

A 32-year-old male with no reported past medical history presented to the hospital after sustaining a gunshot wound to the left thigh by an unknown assailant. On the primary survey, his examination was notable for normal blood pressure of 121/77 mmHg, tachycardia to 109 beats per minute, and decreased 1+ pedal pulses in the affected extremity. A secondary survey revealed a gunshot wound to the left lateral thigh and another to the left medial thigh, consistent with a through-and-through injury. Mild swelling of the left thigh and foot was also observed. By the time of the secondary survey, approximately two minutes after the initial assessment, the patient’s pulse examination had normalized without vascular intervention, with 2+ pedal pulses bilaterally. The remainder of the examination demonstrated neither hard nor soft signs of arterial injury (Table 1).

**Table 1:** Clinical signs of peripheral vascular injury Summary of clinical hard and soft signs associated with peripheral vascular injury, adapted from published guidelines [1,2].

Hard signs	Soft signs
Pulsatile bleeding	Nonexpanding/nonpulsatile hematoma
Expanding/pulsating hematoma	Diminished pulse
Loss of distal pulses	Proximity of the wound to an artery
Bruit/thrill	Neurologic deficit

Doppler occlusion pressures of the dorsalis pedis artery were measured in both lower extremities and were 142 mmHg in the affected left extremity and 150 mmHg in the right extremity, yielding an arterial-arterial index of 0.95 (i.e., within the normal range). Although CTA was not indicated based on current guidelines (Table 2), the decision was made to obtain computed tomographic angiography of the left lower extremity because of the edema.

**Table 2:** EAST recommendations for the evaluation and management of penetrating lower extremity vascular injury Summary of evidence-based recommendations from the EAST for the evaluation and management of penetrating lower extremity vascular injury, including the use of computed tomographic angiography, criteria for surgical exploration, and the role of the ABI in guiding further assessment or discharge. Adapted and paraphrased from published guidelines [1]. ABI, ankle-brachial index; EAST, Eastern Association for the Surgery of Trauma

Level	Recommendation
1	Computed tomographic angiography is recommended as the initial imaging modality for evaluating suspected penetrating vascular injuries of the lower extremity when diagnostic imaging is indicated.
2	(1) Patients presenting with hard signs of arterial injury (e.g., pulse deficit, pulsatile hemorrhage, bruit, thrill, or expanding hematoma) should proceed directly to surgical exploration. Arteriography is not required unless there is a concurrent skeletal or shotgun injury. Revascularization should ideally occur within 6 hours to optimize limb salvage. (2) In the absence of hard signs, patients with abnormal physical examination findings or an ABI <0.9 should undergo further evaluation to assess for vascular injury. (3) Patients with normal physical exams and an ABI >0.9 may be considered for discharge if no other injuries warrant admission.

**Figure 1:**
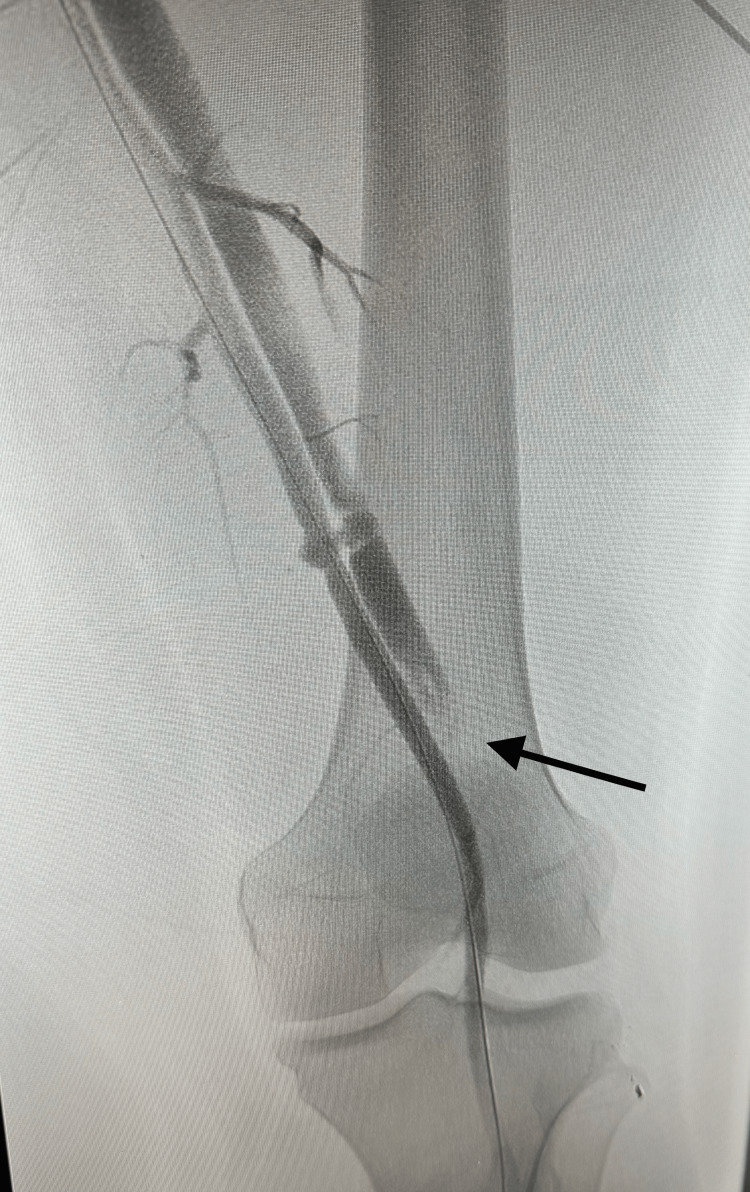
Pre-intervention angiogram demonstrating traumatic femoral AVF A still image from the fluoroscopic angiogram of the left lower extremity performed on the day of presentation, prior to intervention. The image is oriented in the anteroposterior projection. Early filling of the femoral vein (black arrow) is observed, indicating arteriovenous shunting consistent with a traumatic femoral AVF. AVF, arteriovenous fistula

The computed tomographic angiography demonstrated early filling of the femoral vein, consistent with a traumatic AVF of the femoral vessels in the mid-left thigh (Figure 1), and vascular surgery was consulted for management.

**Figure 2:**
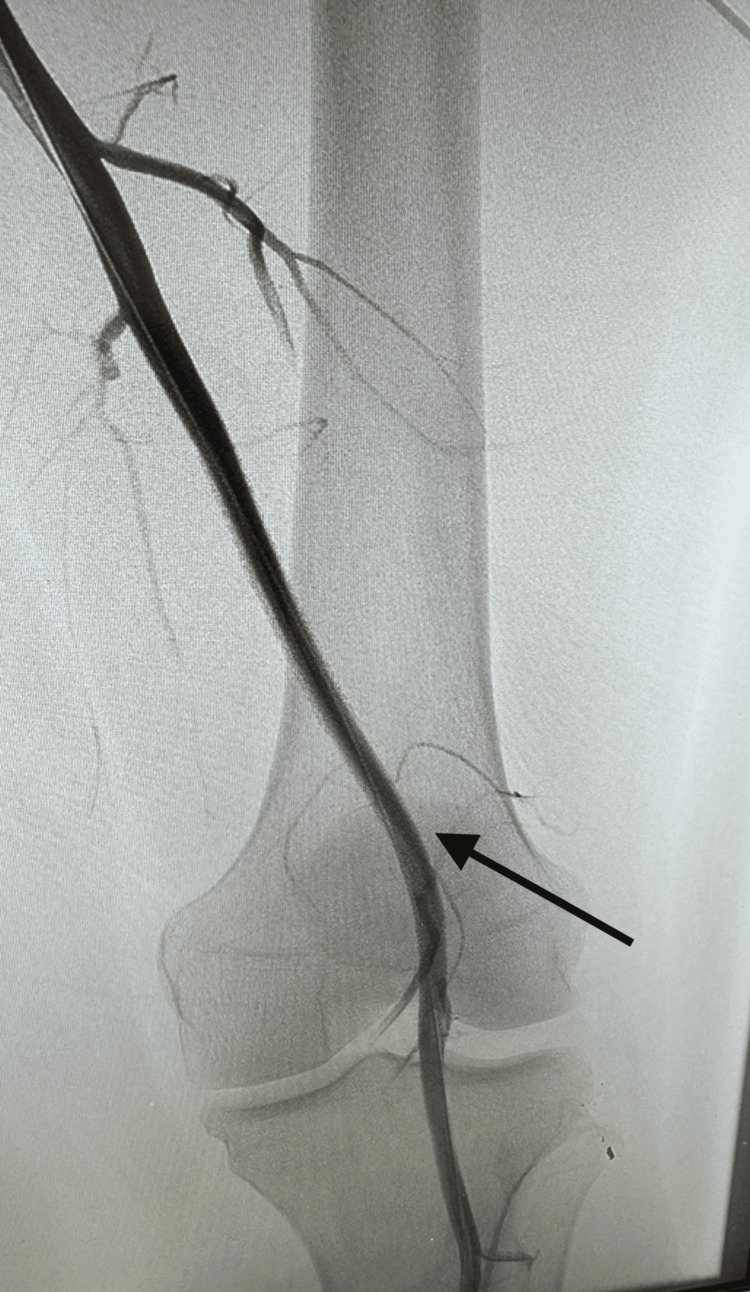
Post-intervention angiogram demonstrating resolution of traumatic femoral AVF following stent placement Angiogram of the left lower extremity following covered stent placement in the superficial femoral artery. The image is oriented in the anteroposterior projection and demonstrates resolution of early venous filling with restoration of normal arterial flow (black arrow) after stent placement for traumatic femoral AVF. AVF, arteriovenous fistula

The traumatic AVF was confirmed with duplex sonography, and the patient was taken to the cath lab the following morning for angiography and treatment with a covered stent, which successfully resolved the traumatic AVF (Figure 2). Postoperatively, the patient had equal 2+ pedal pulses bilaterally and was discharged on postoperative day zero with aspirin and clopidogrel. He was scheduled for follow-up with the vascular clinic for ongoing management. At his one-month postoperative clinic visit, he was found to be doing well, with palpable bilateral pulses and no ischemic symptoms. He was scheduled for repeat vascular laboratory studies one month later as part of routine surveillance; however, he was unfortunately lost to follow-up. 

## Discussion

Missed vascular injuries can lead to serious complications, including pseudoaneurysm, neuropathy, skin ulceration, thromboembolic events, limb loss, fistula rupture with hemorrhage, and congestive heart failure [6,9,10]. While CTA is a valuable diagnostic tool for identifying such injuries, it is not without limitations, including radiation exposure [11]. Therefore, the decision to pursue CTA should be guided by a thorough clinical assessment and integration of all available evidence. Current guidelines recommend determining the need for CTA in patients with penetrating extremity trauma based on the presence of hard or soft signs of vascular injury and the ABI [1,2]. However, our case highlighted a significant limitation of these criteria.

Given the underlying vascular mechanisms, we suggest that unexplained distal edema may warrant consideration as a soft sign prompting vascular imaging. In cases of penetrating extremity trauma, distal edema not attributable to a preexisting medical condition should raise suspicion for vascular compromise and prompt further evaluation, such as CTA. This recommendation is supported by the pathophysiology of a traumatic AVF. An AVF creates an abnormal connection between an artery and a vein, significantly increasing blood flow in the affected region [5]. The resulting surge in flow and pressure overwhelms the venous system’s capacity to drain blood effectively, leading to interstitial fluid accumulation and distal edema. Furthermore, the diversion of arterial blood directly into the venous system causes venous hypertension, which further impairs normal venous return [12].

Although trauma-induced inflammatory responses can also produce localized swelling near the injury site, this process is distinct from vascular compromise because it does not elevate venous pressure. It is essential to differentiate focal post-traumatic swelling from circumferential distal edema, which is more indicative of significant vascular impairment. In this context, the presence of distal edema following a penetrating injury should be considered a strong indicator of vascular compromise and warrants urgent evaluation with CTA, even when the ABI is above 0.90. The underlying pathophysiology underscores the need to reassess whether unexplained distal edema should be regarded as a soft sign of vascular injury in trauma protocols.

**Figure 3:**
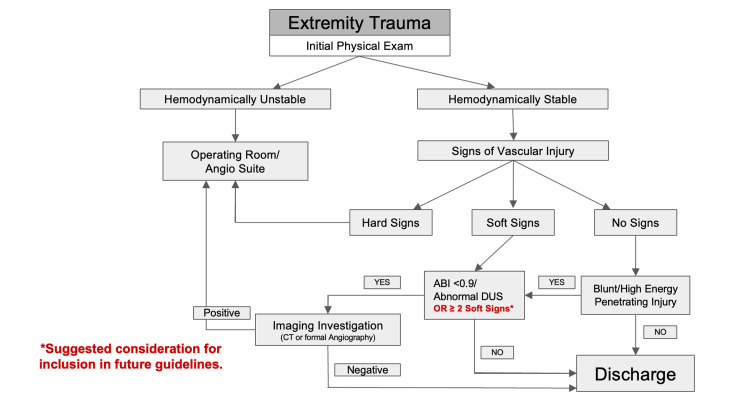
Peripheral vascular injury diagnostic management algorithm Decision tree adapted from the AAST-World Society of Emergency Surgery guidelines on the diagnosis and management of peripheral vascular injuries [2]. AAST, American Association for the Surgery of Trauma; ABI, ankle-brachial index; DUS, duplex ultrasonography

Although formal modifications to existing guidelines should be supported by broader evidence, our case highlighted the potential benefit of considering the cumulative presence of soft signs when evaluating the need for vascular imaging. Specifically, Figure 3 illustrates a conceptual algorithm in which the presence of two or more soft signs, regardless of ABI, could prompt consideration of CTA. This approach is consistent with prior studies reporting vascular injury rates as high as 25% in patients with multiple soft signs [13,14]. In our case, the patient exhibited both a history of arterial bleeding and a diminished pulse in the affected extremity at presentation. While further research is needed, this model may provide a more nuanced framework for imaging decisions in select trauma patients.

## Conclusions

This case underscores that clinically significant vascular injuries, such as traumatic femoral AVF, can occur despite normal physical examination findings and an ABI greater than 0.90. In our patient, unexplained distal limb edema was the only subtle clue that prompted further imaging, ultimately leading to timely diagnosis and treatment. We propose that distal edema be recognized as a potential soft sign of vascular injury in extremity trauma and that its presence, particularly in combination with other soft signs, should prompt consideration of CTA regardless of ABI. Early recognition and intervention are critical to preventing severe complications, including limb loss, thromboembolism, and hemorrhage. Incorporating this refinement into existing trauma protocols may enhance diagnostic sensitivity, support more efficient use of imaging resources, and ultimately improve patient outcomes.
